# Cannabinoid receptor-1 inhibition causes anesthetic-induced excitation in septic rats

**DOI:** 10.1186/cc9780

**Published:** 2011-03-11

**Authors:** R Kuschnereit, C Lehmann, S Whynot, O Hung, R Shukla, D Henzler, V Cerny, D Pavlovic, M Kelly

**Affiliations:** 1 Dalhousie University, Halifax, Canada

## Introduction

In systemic inflammation and sepsis, the endo-cannabinoid system is upregulated [1]. While it is known that neuronal cannabinoid signalling via cannabinoid receptor-1 (CB1) in the central nervous system represents an intrinsic neuroprotective response [2] and exerts anti-epileptic activity [3], inhibition of CB1 (CB1inh) has been suggested as an experimental target for sepsis therapy [4]. We studied the effects of CB1inh in rats with experimental sepsis during anesthesia induction with pentobarbital.

## Methods

Five groups of Lewis rats were included in the study: Group 1 - sham-operated controls treated with CB1inh (AM281, 2.5 mg/kg i.v., *n *= 12), Group 2 - animals with colon ascendens stent peritonitis (CASP)-induced sepsis treated with CB1inh (*n *= 12). As additional control groups we administered in CASP animals the CB1 agonist ACEA (2.5 mg/kg i.v.; Group 3; *n *= 4) or the solvent DMSO (Group 4; *n *= 4). In Group 5 we administered 50 mg/kg ketamine for induction of anesthesia 14 hours following the CASP treated by CB1inh. All other groups received a standard dose of pentobarbital (40 mg/kg i.v.) 14 hours following CASP procedure.

## Results

In five out of 12 septic animals (42%) with CB1inh (Group 2) we observed tonic-clonic seizures immediately after induction of anesthesia with a standard dose of pentobarbital. In sham-operated animals (Group 1) or CASP animals without CB1inh (Group 4) we did not observe anesthetic-induced excitation. Replacement of the barbiturate by ketamine (Group 5) avoided seizures as well as treatment with the CB1 agonist (Group 3).

## Conclusions

CB1 inhibition in sepsis may increase the incidence of anesthetic-induced excitation and reduce CB1-mediated intrinsic neuroprotective response.
